# Changes in clinical laboratory parameters and pharmacodynamic markers in response to blinatumomab treatment of patients with relapsed/refractory ALL

**DOI:** 10.1186/s40164-017-0074-5

**Published:** 2017-05-18

**Authors:** Virginie Nägele, Andrea Kratzer, Gerhard Zugmaier, Chris Holland, Youssef Hijazi, Max S. Topp, Nicola Gökbuget, Patrick A. Baeuerle, Peter Kufer, Andreas Wolf, Matthias Klinger

**Affiliations:** 1 Amgen Research (Munich) GmbH, Staffelseestrasse 2, 81477 Munich, Germany; 2 Amgen Inc., Rockville, MD USA; 3 Medizinische Klinik und Poliklinik II, Universitätsklinikum Würzburg, Würzburg, Germany; 4 Department of Medicine II, Goethe University, Frankfurt, Germany

**Keywords:** Acute lymphoblastic leukemia, Blinatumomab, Bispecific, BiTE^®^, CD19, Liver enzymes, Pharmacodynamics

## Abstract

**Background:**

Blinatumomab has shown a remission rate of 69% in an exploratory single-arm, phase II dose-escalation study in adult patients with relapsed/refractory B-precursor acute lymphoblastic leukemia (ALL). We evaluated changes in laboratory parameters and immunopharmacodynamic markers in patients who received blinatumomab in the exploratory phase II study.

**Methods:**

Data from 36 adults with relapsed/refractory ALL receiving blinatumomab as 4-week continuous IV infusions in various dose cohorts were analyzed for changes in liver enzymes, first-dose parameters, peripheral blood cell subpopulations, and cytokine/granzyme B release. Associations with clinical response were evaluated.

**Results:**

Liver enzymes and inflammatory parameters transiently increased primarily during the first treatment week without clinical symptoms and reversed to baseline levels thereafter. B and T cells showed expected depletion and redistribution kinetics, respectively. Similarly, thrombocytes and T cells displayed an initial decline in cell counts, whereas neutrophils peaked during the first days after infusion start. T-cell redistribution coincided with upregulation of LFA-1 and CD69. Patients who responded to blinatumomab had more pronounced T-cell expansion, which was associated with proliferation of CD4^+^ and CD8^+^ T cells and memory subsets. Release of cytokines and granzyme B primarily occurred during the first week of cycle 1, except for IL-10, which was released in subsequent cycles. Blinatumomab step-dosing was associated with lower cytokine release and lower body temperature.

**Conclusions:**

In this study of relapsed/refractory ALL, blinatumomab-induced changes in laboratory parameters were transient and reversible. The evaluated PD markers demonstrated blinatumomab activity, and the analysis of cytokines supported the rationale for stepwise dosing.

(*ClinicalTrials.gov Identifier* NCT01209286.)

**Electronic supplementary material:**

The online version of this article (doi:10.1186/s40164-017-0074-5) contains supplementary material, which is available to authorized users.

## Background

The CD19/CD3 bispecific T-cell engager (BiTE^®^) antibody construct blinatumomab is an adaptor protein that allows T cells to recognize specifically CD19-expressing B cells [1], thereby directing the cytotoxic potential of the T cell towards the targeted B cell. Numerous preclinical studies have demonstrated this mode of action, showing complete target cell lysis at very low blinatumomab concentrations and effector-to-target cell ratios along with tumor eradication in xenograft models [2]. Clinical response to blinatumomab treatment has been evaluated in relapsed/refractory non-Hodgkin’s lymphoma (NHL), relapsed/refractory acute lymphoblastic leukemia (ALL), and minimal residual disease (MRD)-positive ALL [3–8]. In an exploratory phase II dose-finding study in relapsed/refractory ALL, 69% of patients achieved complete remission (CR) or CR with partial hematologic recovery (CRh) within two treatment cycles. The study identified the blinatumomab target dose as 15 µg/m^2^/day using a 1-week run-in phase at 5 µg/m^2^/day for mitigation of first-dose effects [6]. In long-term follow-up analysis, T-cell expansion was associated with long-term survival [9]. In the subsequent large confirmatory phase II study, 43% of patients with relapsed/refractory ALL achieved CR/CRh within the first two cycles of blinatumomab treatment [7].

The first comprehensive pharmacokinetic (PK) and pharmacodynamic (PD) analysis in response to blinatumomab treatment was conducted in patients who were in complete hematologic remission after receiving treatment for ALL but maintained MRD-positive disease, an indicator of chemotherapy resistance [10]. T-cell and B-cell distribution kinetics and markers of blinatumomab mode of action in patients with relapsed/refractory ALL, an aggressive disease with a very poor prognosis [11, 12], have not yet been studied. However, evaluating blinatumomab-induced PD effects in this setting is an important first step in elucidating potential biomarkers for clinical outcomes. Furthermore, PD analyses may contribute to the management of adverse events associated with the blinatumomab mode of action. For example, treatment-induced cytokine release may cause rare events of cytokine release syndrome (CRS), and blinatumomab treatment has been associated with changes in liver enzyme parameters [6, 7]. Medications or factors related to hepatic injury/dysfunction and cholestasis or biliary obstruction may cause liver enzyme elevations above normal levels even in otherwise healthy individuals [13–16]. Thus, detailed serum chemistry, including liver enzymes such as alkaline phosphatase (AP), alanine aminotransferase (ALT), aspartate aminotransferase (AST), total bilirubin, and gamma-glutamyl transferase (GGT), provides important information on a patient’s liver function in response to drug treatment and may reveal drug-induced hepatocellular, cholestatic or mixed liver injury [17–19].

In the present study, we analyzed for the first time changes in liver enzymes and markers of inflammation and coagulation in response to blinatumomab treatment administered to patients with relapsed/refractory ALL in the exploratory phase II dose-finding study. Furthermore, we performed a detailed assessment of the behavior of peripheral T and B cells, neutrophils, and thrombocytes and characterized the release of cytokines and the T-cell effector molecule granzyme B.

## Methods

### Patients

Detailed inclusion/exclusion criteria are published elsewhere [6]. Briefly, adult patients with relapsed/refractory ALL were eligible if they expressed the B-precursor phenotype and had >5% leukemic blasts in the bone marrow. Relapse was defined as reappearance of disease after CR of 28-day duration; refractory disease was defined as not having achieved CR after induction and/or consolidation treatment. A total of 36 patients were enrolled and treated with blinatumomab. ClinicalTrials.gov identifier: NCT01209286.

### Study design

Study design and dose cohorts are described in detail elsewhere [6]. Briefly, this was an open-label, multicenter, phase II study with Simon 2-stage design investigating the efficacy, adverse events, PK, and PD of blinatumomab in patients with relapsed/refractory ALL. Patients received blinatumomab continuous IV infusion at a flat dose of 15 µg/m^2^/day (*n* = 7; cohort 1), or a stepwise dose of 5‒15 µg/m^2^/day (5 µg/m^2^/day for the first 7 days and 15 µg/m^2^/day thereafter; *n* = 5 in cohort 2a; *n* = 18 in extension cohort 3) or 5‒15‒30 µg/m^2^/day (as in cohort 2a with an additional dose step to 30 µg/m^2^/day in week 3; *n* = 6; cohort 2b) over 4 weeks followed by a 2-week treatment-free period (one cycle). Patients who achieved CR or CRh within the first two cycles could receive up to three additional treatment cycles (induction and consolidation). The core study period included screening plus the treatment period (up to five cycles).

### Response measurement

The primary endpoint was achievement of CR or CRh within the first two treatment cycles. CR was defined as bone marrow blasts ≤5%, no evidence of disease, and full recovery of peripheral blood counts (platelets >100,000/µL, hemoglobin [Hb] ≥11 g/dL, absolute neutrophil count [ANC] >1500/µL); CRh was defined as bone marrow blasts ≤5%, no evidence of disease, and partial recovery of peripheral blood counts (platelets >50,000/µL, Hb ≥7 g/dL, ANC >500/µL). Bone marrow blast count was quantified by a central laboratory at screening and after each treatment cycle.

### Anti-blinatumomab antibodies

Serum samples for detection of anti-blinatumomab antibodies were collected at baseline (predose), at the end of infusion of each treatment cycle, and at the end-of-core-study visit. Anti-blinatumomab antibodies were measured with a validated electrochemiluminescence immunoassay (Meso Scale Discovery, Rockville, MD, USA). Briefly, serum samples (undiluted and prediluted 1:100 in the respective predose serum in order to avoid potential hook effects) were diluted 1:10 in phosphate-buffered saline and then incubated with 0.5 µg/mL each of biotin- and ruthenium-conjugated blinatumomab (prepared using MSD SULFO-TAG™ [Meso Scale Discovery] following the manufacturer’s instructions) for at least 1 h at room temperature. Samples were then added to a streptavidin-coated 96-well microtiter plate (Meso Scale Discovery) blocked with 5% bovine serum albumin in phosphate-buffered saline at room temperature and incubated for 0.5–2 h to allow formation of antibody complexes. Anti-blinatumomab antibodies in patient serum bound to biotin-conjugated/streptavidin-captured blinatumomab were recognized by ruthenium-conjugated blinatumomab. After washing with phosphate-buffered saline plus 0.05% Tween 20 and application of Reading Buffer (Meso Scale Discovery), signals were measured using a Sector Imager 2400 analyzer (Meso Scale Discovery) and normalized against a predose serum sample tested in parallel. Polyclonal goat anti-blinatumomab antibodies (Biogenes, Berlin, Germany) were included as positive control. Positive serum samples were retested in a competitive inhibition assay determining percent change in assay signal with and without blinatumomab preincubation.

### Pharmacokinetics

Blood samples were collected before, during, and after infusion: baseline (day 1), days 3, 8, 15, 22, and 29 in all cohorts; and at additional time points (day 8 + 2 h, day 8 + 6 h; days 9, 10, and 17) in some cohorts. Biologically active concentrations of blinatumomab in serum were analyzed as described previously [20], using an assay based on CD69 upregulation on the surface of newly activated T cells after dual binding of blinatumomab to HPB-ALL T cells and Raji B-lymphoma cells. The dose-dependent increase of CD69 expression was measured using a fluorescence-activated cell sorter (FACS) instrument (FACSCalibur or FACSCanto II; BD Biosciences, Heidelberg, Germany). Data were analyzed using GraphPad Prism 6 software (GraphPad Software, La Jolla, CA, USA) or SoftMax Pro software (MDS Analytical Technologies, Sunnyvale, CA, USA). The assay was internally validated; the lower limit of quantification (LLOQ) was 50 pg/mL. The mean steady state concentration (C_ss_) of blinatumomab in serum from individual patients was calculated from available data points at exposure plateau in each 4-week treatment period. For each dose cohort, the data from individual cycles were included as independent data points.

### Serum chemistry

Blood samples for evaluation of clinical laboratory parameters were collected during the screening period (day −21 to day 0), before treatment start (baseline [day 1]), and during treatment (days 2, 3, 8, 15, 22, 29) for up to 5 cycles. AST, ALT, GGT, lactate dehydrogenase (LDH), total bilirubin, and C-reactive protein (CRP) were analyzed using samples from all time points. Patient inclusion criteria were <5× upper limit of normal (ULN) for AST, ALT or AP and <1.5 × ULN for total bilirubin [7]. Numbers of white blood cells and thrombocytes were determined from differential blood count. D-dimer concentrations were measured only in samples collected during the first two treatment cycles. No threshold values for treatment discontinuation were defined for the laboratory parameters.

### Lymphocyte subpopulations

Lymphocyte subpopulations were measured either in density gradient-separated peripheral blood mononuclear cells, prepared as described previously [10], or in whole peripheral blood at screening, before treatment start (baseline), and at various time points during the infusion periods as well as at the end of the core study and at follow-up visits. Briefly, peripheral blood was collected more frequently in cohorts 1 and 2a: within 1 h before and at 2, 6, 24, and 48 h after treatment start and dose step, if any, and again once weekly until end of infusion. In cohorts 2b and 3, peripheral blood was only collected within 1 h before and 48 h after treatment start and dose step(s), and again once weekly until end of infusion. Lymphocyte subpopulations were analyzed by flow cytometric determination of different cell surface markers, obtaining measures of the relative cellular composition of the blood sample using an eight-color FACSCanto II instrument (BD Biosciences), a five-color FC500 instrument (Beckman Coulter, Brea, CA, USA), or a ten-color FACS NAVIOS instrument (Beckman Coulter). Fluorescent dye-labelled monoclonal antibodies were used to detect the following cell surface markers: CD45 (lymphocytes); CD19 (B cells); CD3, CD4, CD8 (T cells); CD69 (T-cell activation); CD45RA, CD28, CCR7 (memory T-cell subsets). T-cell adhesiveness was assessed by binding of soluble ICAM-1-Fc fusion proteins (R&D Systems, Abingdon, UK) to (activated) LFA-1 on T cells, with subsequent detection by goat anti-human IgG, Fc-FITC (Dianova, Hamburg, Germany). By combining percentage values of certain lymphocyte subpopulations with the absolute lymphocyte number determined by differential blood count, the absolute numbers of the respective subpopulations were calculated.

### Cytokines and granzyme B

Peripheral blood cytokine levels of interleukin (IL)-2, IL-4, IL-6, IL-10, TNF-α, and IFN-γ were monitored by measuring the respective markers in serum using the FACS-based BD Cytometric Bead Array Human Th1/Th2 Kit II (BD Biosciences) following the manufacturer’s instructions. Signals were measured using a FACSCanto II instrument and the FACS Diva evaluation software (BD Biosciences). Cytokine concentrations were calculated with the FCAP array software (Soft Flow Inc., St Louis Park, USA). The assay was internally validated; the LLOQ was 125 pg/mL and the limit of detection (LOD) was 20 pg/mL. Serum samples were collected approximately 1 h before infusion start (baseline [day 1]); at 2, 6, 24, 48, and 168 h after treatment start; and at these same time points after each dose step (if applicable) in each treatment cycle for up to five cycles. For calculations of mean cytokine peak concentrations (C_max_) across all patients who received a starting dose of 5 or 15 µg/m^2^/day during treatment week 1, or 15 µg/m^2^/day as second dose during treatment week 2, and for calculations of mean and standard deviation of cytokine levels per cycle, values below LLOQ were included as such; values below LOD were set to ½ LOD to allow logarithmic plotting. Restarted cycles following treatment interruption were considered as new cycles.

Granzyme B was monitored in parallel to cytokines in treatment cycle 1. Serum samples were collected at the time points described above. Granzyme B was measured in triplicate using the Human Granzyme B Platinum ELISA kit (eBioscience, San Diego, USA). The assay was internally validated with an LLOQ of 100 pg/mL and an LOD of 30 pg/mL. Calculations of mean granzyme B peak concentrations and mean granzyme B levels per cycle were performed as described above.

### Body temperature

Peak body temperature was measured before treatment start (baseline); every 4 h within the first 12 h of infusion start; in the morning and evening on treatment day 2 and 3; and once on day 8, 15, 22 and 29. Peak body temperature was analyzed for the first treatment week in patients who received a starting dose of 5 or 15 µg/m^2^/day in cycle 1 and for the second treatment week in patients who received 15 µg/m^2^/day as the second dose in cycle 1.

### Statistical analysis

All data were summarized using descriptive statistics. Data are presented as mean ± standard deviation (SD) or as median with 25th and 75th percentile.

## Results

### Pharmacokinetics and anti-blinatumomab antibodies

In this exploratory phase II study, 36 patients with relapsed/refractory ALL received blinatumomab via continuous IV infusion. In cycle 1, treatment was administered over 4 weeks either at a flat (15 µg/m^2^/day) or stepwise dose (5‒15 or 5‒15‒30 µg/m^2^/day) [6]. The mean (±SD) C_ss_ of blinatumomab in serum across all patients with available samples was 198 (±61) pg/mL at the step dose of 5 µg/m^2^/day (*n* = 20) and 694 (±236) pg/mL at 15 µg/m^2^/day (*n* = 32).

Paired pre- and post infusion blood samples were collected from 35 of the treated 36 patients (no post infusion sample was available for one patient). No anti-blinatumomab antibodies were detected in any of the samples.

### Transient blinatumomab-induced changes in clinical laboratory parameters

**Fig. 1:**
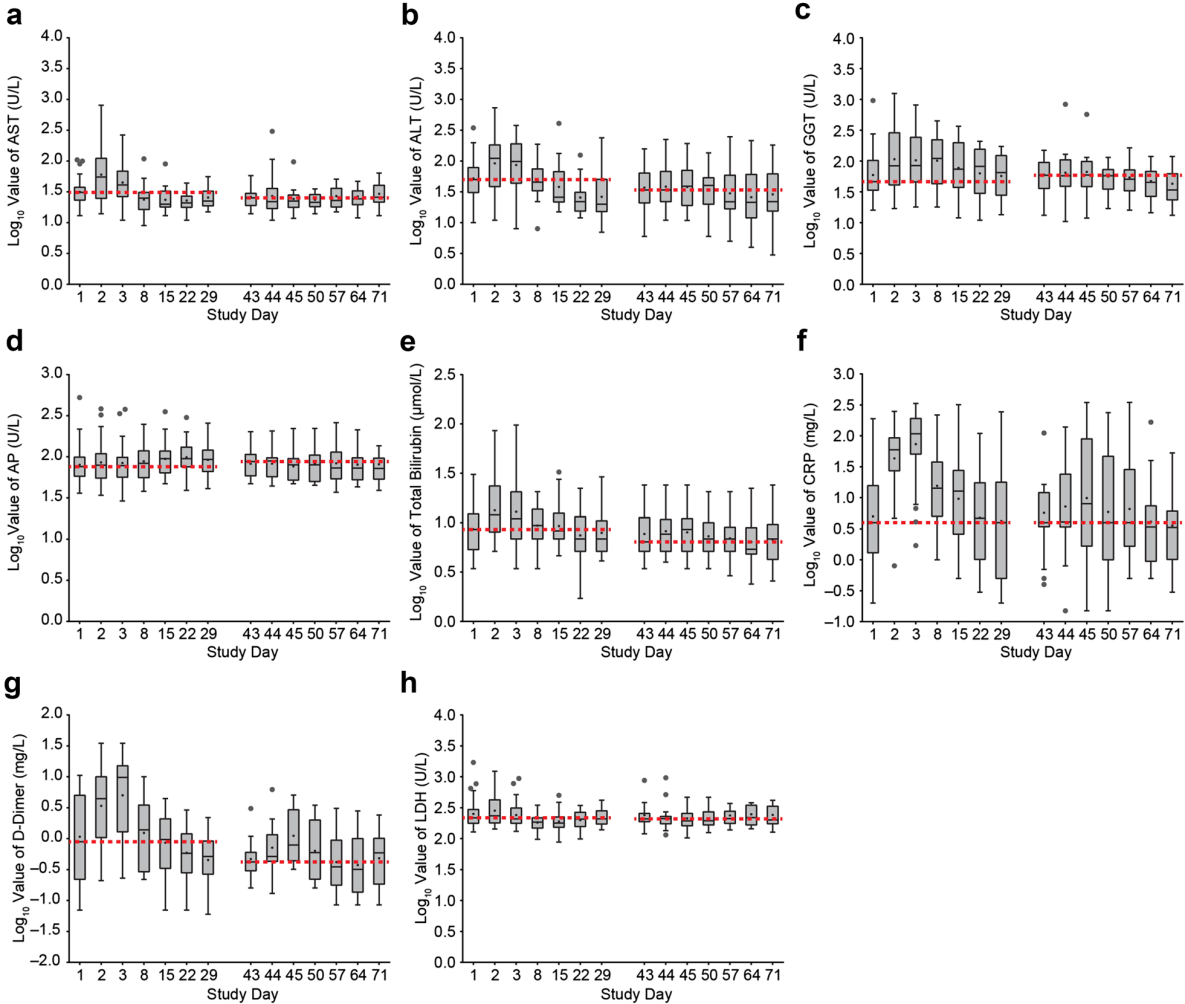
Liver and first-dose parameters transiently increase primarily during the first treatment week of cycle 1. Box plots depicting logarithmic serum values of aspartate aminotransferase (AST) (**a**), alanine amino transferase (ALT) (**b**), gamma glutamyl transferase (GGT) (**c**), alkaline phosphatase (AP) (**d**), total bilirubin (**e**), C-reactive protein (CRP) (**f**), D-dimer (**g**), and lactate dehydrogenase (LDH) (**h**) in all evaluable patients during cycle 1 and 2. *Black line* median levels; *plus sign* mean levels; *circle* outlying value; *red dashed line* baseline level in cycle 1 or cycle 2. *Boxes* extend from the 25th to 75th percentile, with *bars* extending to the minimum and maximum values within 1.5 times the interquartile range (difference between the 25th and 75th percentile). Corresponding patient numbers are shown in Additional file 1

We monitored changes in clinical laboratory parameters by analyzing serum chemistry, hematology, and coagulation over the course of blinatumomab treatment. Liver parameters showed elevated median levels of AST, ALT, and total bilirubin within 24 h and elevated GGT within 1 week after infusion start, followed by a decline to baseline levels by end of cycle 1 (Fig. 1a–c, e). During cycle 1, the ULN range [19] was exceeded for AST (1.6-fold; normal range, 0‒35 U/L), ALT (3.2-fold; normal range, 0‒35 U/L), and GGT (1.3-fold; normal range, 9‒85 U/L). After the 2-week treatment-free interval, median peak levels of total bilirubin and GGT were reached within 48 h and median peak levels of ALT within 1 week after infusion restart (Fig. 1b, c, e). Median levels of AST remained almost stable throughout the second treatment cycle (peak maximum on day 71) (Fig. 1a). However, the magnitude of median peak levels for ALT and total bilirubin during cycle 2 was lower than during cycle 1. In contrast, median levels of AP did not markedly change during both cycles (peak maxima on day 15/22 and day 44) (Fig. 1d) and remained, as did bilirubin, below the ULN range (41‒133 U/L and 0.1‒1.2 mg/dL, respectively) [19].

Analysis of CRP and D-dimer concentrations during cycle 1 showed similar time courses, with initial elevation of median levels after infusion start, maximum concentrations at 48 h, and a return to baseline at the end of cycle 1. Peak median levels were higher than the ULN range for both CRP (up to 10.8-fold; normal range, <1.0 mg/dL) and D-dimer (up to 19.6-fold; normal range, <500 ng/mL) [19] (Fig. 1f, g). Elevations of median CRP and D-dimer levels in cycle 2 were less pronounced than in cycle 1. Peak median LDH levels were also observed 24 h after infusion start (1.02-fold above the ULN range of 88‒230 U/L) [19]; thereafter LDH levels did not markedly change (peak maximum at day 64) (Fig. 1h). No patients interrupted or discontinued treatment because of elevated liver or first-dose parameters (data not shown).

### Blinatumomab-induced changes in peripheral blood cell subpopulations

Time courses of neutrophils, thrombocytes, T cells, and B cells during blinatumomab treatment were stratified by responders and nonresponders depending on CR/CRh response within two treatment cycles. For this analysis, 25 of the 36 patients were classified as responders and 11 as nonresponders (including two patients with partial remission and three with hypocellular bone marrow) [6].

**Fig. 2:**
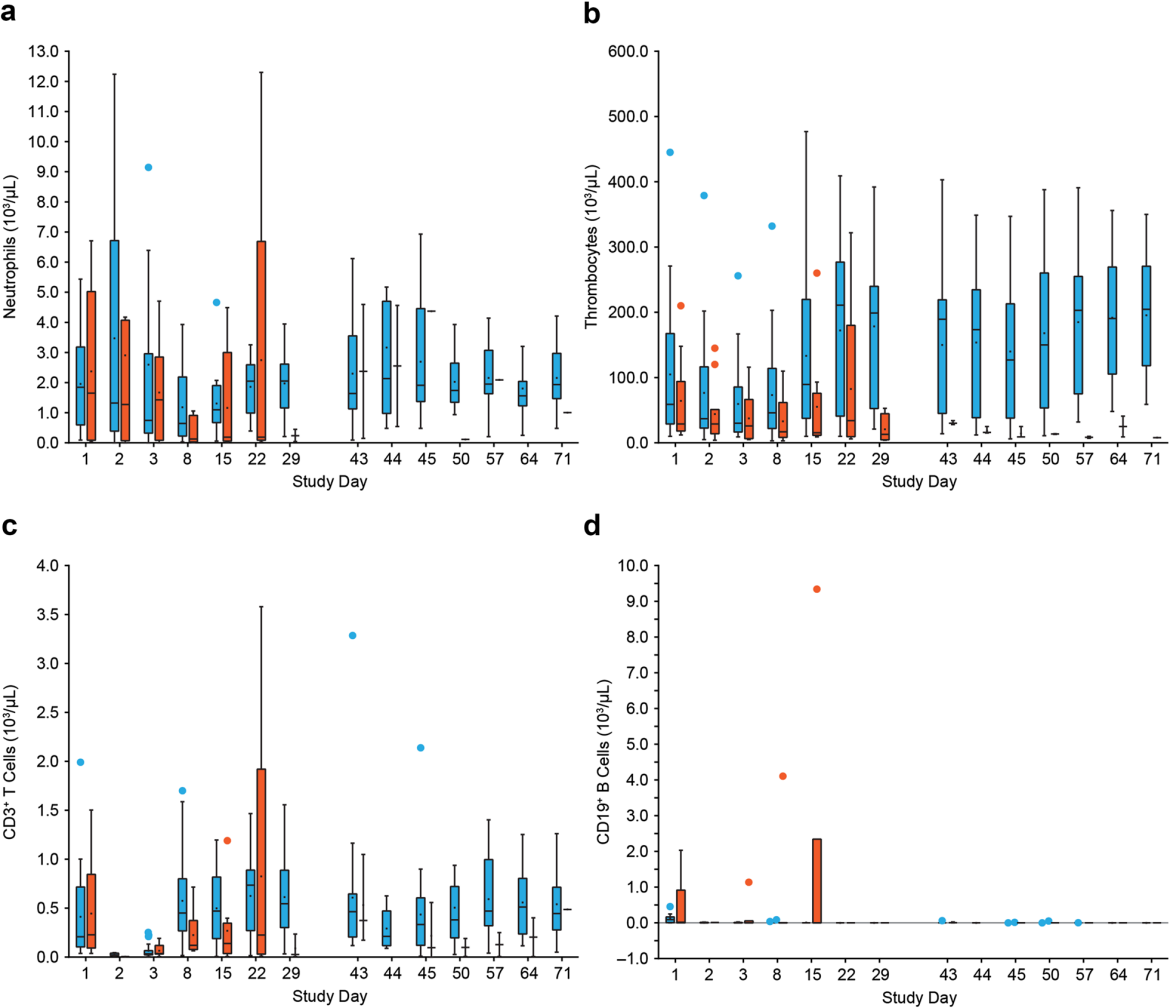
Blinatumomab-induced changes in peripheral blood cell subpopulations. Redistribution of neutrophils (**a**) and thrombocytes (**b**), expansion of T cells (**c**), and kinetics of B-cell depletion (**d**) in responders versus nonresponders during treatment cycle 1 and 2. Patients were stratified into responders (*blue*) and nonresponders (*red*) based on CR/CRh response during the first two treatment cycles.* Box plots* depict cell counts of all evaluable patients during cycles 1 and 2. *Black line* median counts; *plus sign* mean counts; *circle* outlying value. *Boxes* extend from the 25th to 75th percentile, with *bars* extending to the minimum and maximum values within 1.5 times the interquartile range (difference between the 25th and 75th percentile). Corresponding patient numbers are shown in Additional file 2

The median neutrophil count of responders was 1.8 × 10^3^/µL at baseline (Fig. 2a). 24 h after infusion start, the median neutrophil count decreased, reaching a nadir of 0.6 × 10^3^/µL cells after one treatment week before recovering nearly to baseline level at the end of cycle 1 (2.1 × 10^3^/µL). The distribution pattern was different in cycle 2, starting with a median neutrophil count of 1.6 × 10^3^/µL at baseline but reaching a maximum of 2.1 × 10^3^/µL after 24 h before returning to 1.9 × 10^3^/µL at the end of cycle 2. However, comparing the mean neutrophil counts of responders during cycle 1 and 2 showed a clear increase of mean neutrophil numbers after treatment start in both cycles (peak maximum of 3.5 × 10^3^/µL at day 2 in cycle 1 and 3.2 × 10^3^/µL at day 44 in cycle 2).

Median neutrophil counts of nonresponders were 1.7 × 10^3^/µL at baseline, reaching a nadir of 0.1 × 10^3^/µL after 1 week. Recovery did not occur until day 29 of cycle 1 (Fig. 2a). Again, mean neutrophil counts of nonresponders in cycle 1 showed an overall increase after 24 h (from 2.4 to 2.9 × 10^3^/µL). Neutrophil kinetics of nonresponders during cycle 2 could not be analyzed because many nonresponders did not receive a second cycle, resulting in limited data availability.

Thrombocyte redistribution kinetics in responders were characterized by a low initial median thrombocyte count of 5.9 × 10^4^/µL at baseline, reaching a nadir median cell count of 3.0 × 10^4^/µL 48 h after infusion start before recovering to baseline level within 2 weeks (Fig. 2b). The median thrombocyte count of 19.9 × 10^4^/µL at day 29, comparable to that of healthy individuals, corresponded to an increase of 337% above the initial baseline value in cycle 1. After the 2-week treatment-free period, the median thrombocyte count of 19.0 × 10^4^/µL in the responder group remained high for the rest of the second cycle (Fig. 2b). Nonresponders were characterized by thrombocytopenia (initial median cell count of 2.9 × 10^4^/µL). No marked change in thrombocyte counts until day 71 could be detected.

CD3^+^ T cells of responders showed a similar redistribution kinetic during cycle 1, starting with an initial median T-cell count of 2.1 × 10^2^/µL at baseline before reaching a final median count of 5.5 × 10^2^/µL at day 29, which corresponds to a T-cell expansion of 262% (Fig. 2c). The median T-cell count at baseline in cycle 2 was 4.6 × 10^2^/µL, which remained almost stable until day 71 (Fig. 2c). Nonresponders had a similar initial median T-cell count of 2.3 × 10^2^/µL compared with responders, but no measurable expansion over the baseline level during cycle 1.

In contrast to the above differences in peripheral blood cell kinetics between responders and nonresponders, CD19^+^ B cells of both patient groups rapidly declined after infusion start followed by complete B-cell depletion within the first treatment cycle, with the exception of one patient who was nonresponsive to blinatumomab therapy (Fig. 2d). Peripheral B cells of both responders and nonresponders remained undetectable throughout all subsequent treatment cycles (data not shown).

### Blinatumomab-induced T-cell redistribution

**Fig. 3:**
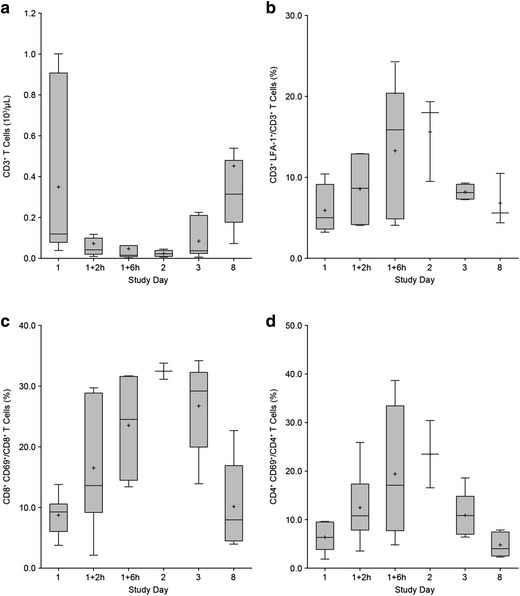
T-cell redistribution during treatment week 1 coincides with activation of LFA-1 and upregulation of CD69. Boxplots depicting the CD3^+^ T-cell counts of patients with frequent blood sampling during the 1st week after start of infusion in cycle 1 (**a**), the percentage of CD3^+^ T cells with activated (i.e., intermediate affinity) LFA-1 (**b**), the percentage of CD8^+^ CD69^+^ (**c**), and CD4^+^ CD69^+^ T cells (**d**). *Black line* median values; *plus sign* mean values. *Boxes* extend from the 25th to 75th percentile, with* bars* extending to the minimum and maximum values within 1.5 times the interquartile range (difference between the 25th and 75th percentile). Corresponding patient numbers are shown in Additional file 3

T-cell redistribution kinetics were studied in more detail for patients who underwent more frequent blood sampling during the first week of blinatumomab infusion (cohorts 1 and 2a). Median CD3^+^ T-cell count was 1.2 × 10^2^/µL at baseline before rapidly decreasing 2 h after infusion start, with a nadir of 0.2 × 10^2^/µL reached after 6 h. After this initial decline, median T-cell counts quickly recovered and even increased above the baseline level within the first treatment week, reaching a median T-cell count of 3.1 × 10^2^/µL at day 8 (264% above baseline) (Fig. 3a). The T-cell redistribution also coincided with upregulation of the higher affinity conformation of the adhesion molecule LFA-1 on CD3^+^ T cells, and with upregulation of the activation marker CD69^+^ on both CD8^+^ and CD4^+^ T cells (Fig. 3b–d). The magnitude of increase from baseline was ~3.6-fold for LFA-1, ~3.5-fold for CD8^+^/CD69^+^, and 3.7-fold for CD4^+^/CD69^+^ T cells, with the peak reached 24 h after infusion start. Values returned to baseline by the end of the first treatment week.

### Blinatumomab-induced T-cell expansion

**Fig. 4:**
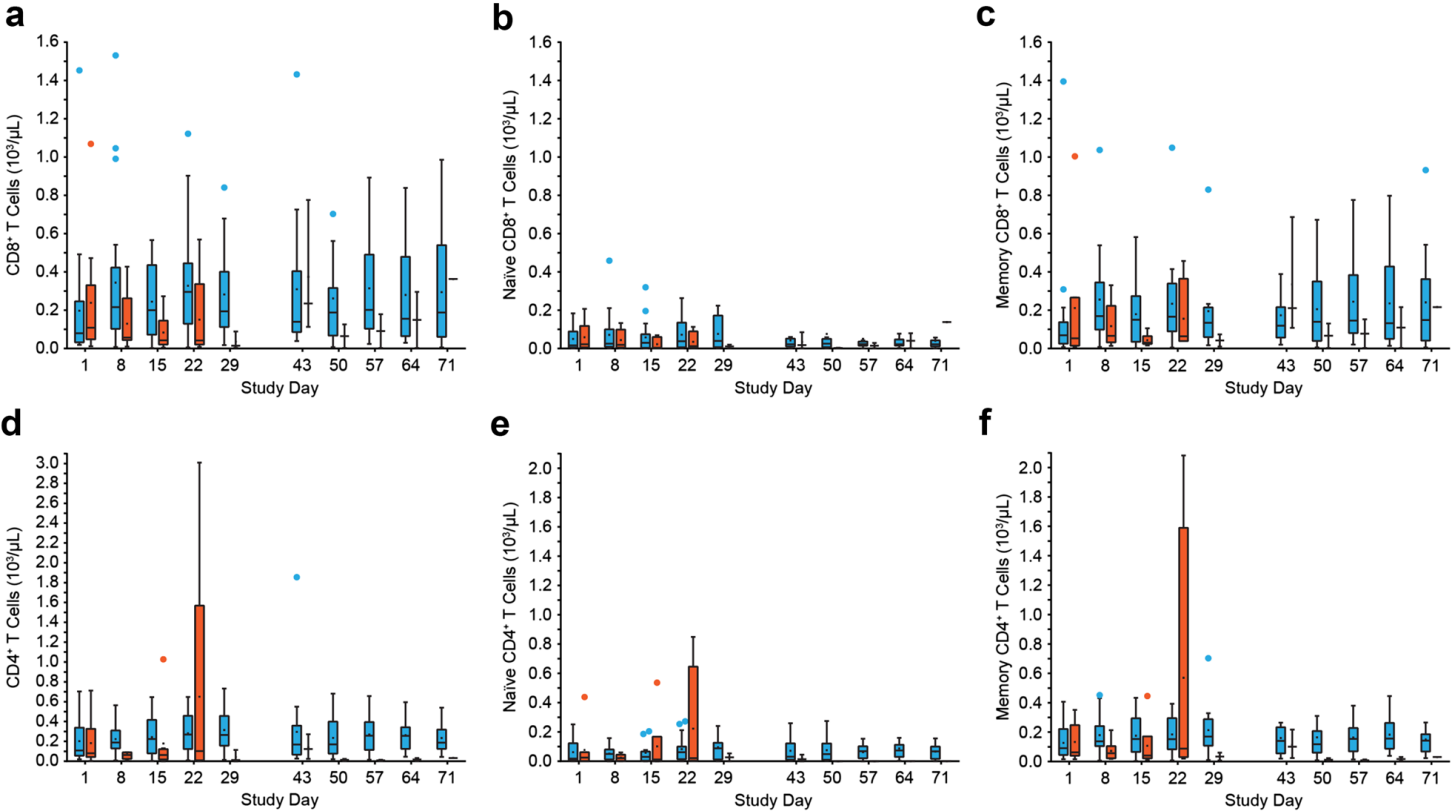
CD8^+^ and CD4^+^ T-cell expansion in responding patients. Patients were stratified into responders (*blue*) and nonresponders (*red*) based on CR/CRh response within the first two treatment cycles.* Box plots* depicting the cell counts of all evaluable patients are shown for CD8^+^ T cells (**a**), naïve CD8^+^ T cells (**b**), memory CD8^+^ T cells (**c**), CD4^+^ T cells (**d**), naïve CD4^+^ T cells (**e**), and memory CD4^+^ T cells (**f**). *Black line* median counts; *plus sign* mean counts; *circle* outlying value. *Boxes* extend from the 25th to 75th percentile, with* bars* extending to the minimum and maximum values within 1.5 times the interquartile range (difference between the 25th and 75th percentile). Corresponding patient numbers are shown in Additional file 4

To examine the contribution of different T-cell subsets to the observed T-cell expansion, we analyzed median cell counts of CD4^+^ and CD8^+^ naïve and memory T-cell subsets during the first and second treatment cycle, and compared its kinetics between responders and nonresponders. Compared with baseline, T-cell expansion of responders was associated with increased median counts of both CD8^+^ T cells and memory CD8^+^ T cells (T_CM_ + T_EM_ + T_EMRA_) at the end of cycle 1 (243 and 195% increase above cycle 1 baseline, respectively) and cycle 2 (134 and 125% increase above cycle 2 baseline, respectively) (Fig. 4a, c). In contrast, median counts of naïve CD8^+^ T cells from responders were lower than CD8^+^ and memory CD8^+^ T cells, exceeding the baseline level only at the end of the first treatment cycle (250% increase; Fig. 4b). CD4^+^ and CD8^+^ T-cell kinetics of responders were comparable. CD4^+^ T cells and memory CD4^+^ T cells (T_CM_ + T_EM_) of responders expanded by 245 and 187%, respectively, above baseline at the end of cycle 1, and by 112 and 102%, respectively, above baseline at the end of cycle 2 (Fig. 4d, f). Median numbers of naïve CD4^+^ T cells in responders were lower than CD4^+^ and memory CD4^+^ T cells, expanding to 489% above baseline at the end of the first treatment cycle and to 216% above baseline at the end of cycle 2 (Fig. 4e). In nonresponders, median counts of CD4^+^ and CD8^+^ T-cell subsets fluctuated around baseline values in cycle 1 without expansion above baseline level (Fig. 4a–f). T-cell distribution profiles of nonresponders during cycle 2 were not evaluable because of low patient numbers.

### Blinatumomab-induced cytokine release

**Fig. 5:**
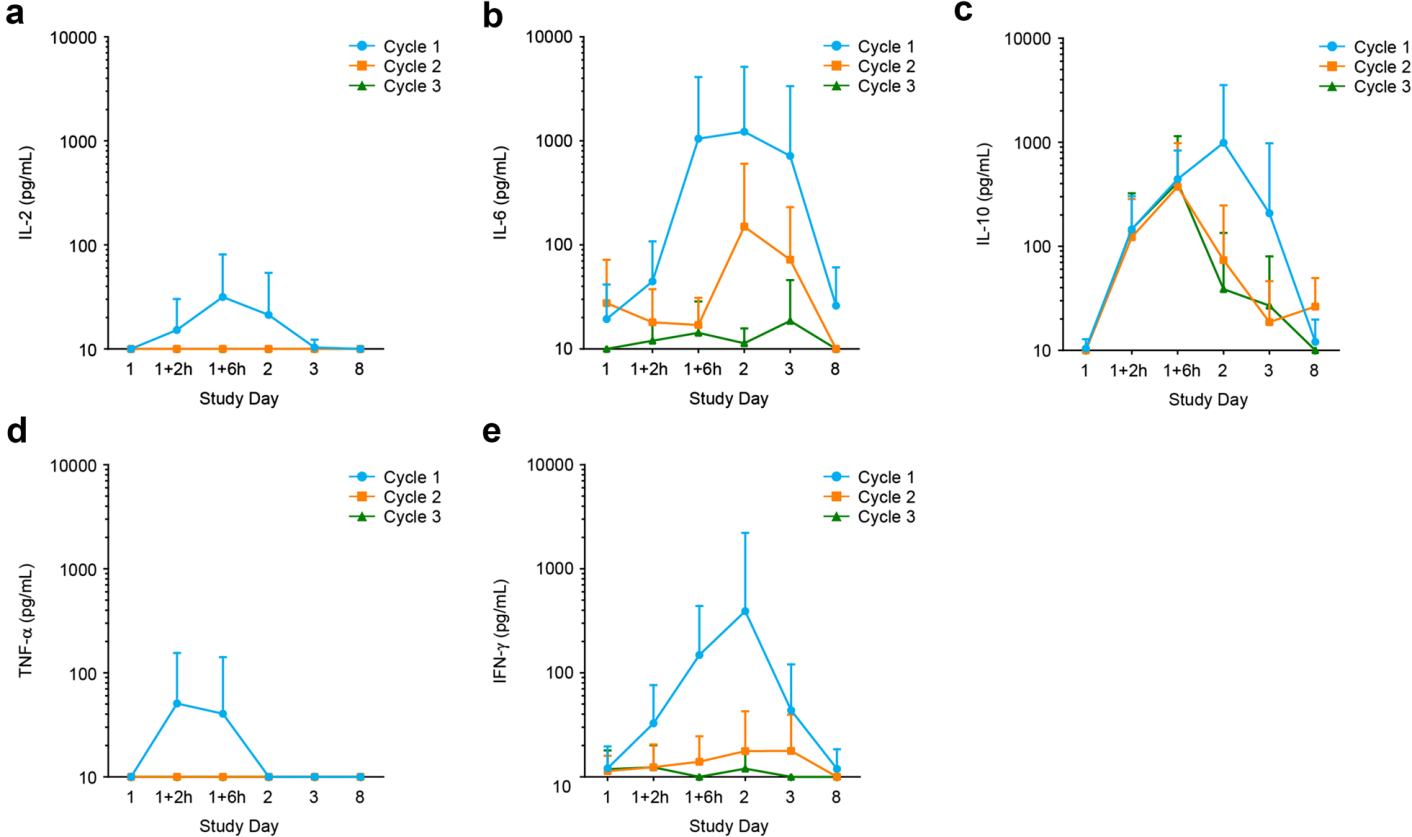
Transient cytokine release primarily occurs during the first days after start of blinatumomab therapy. Mean ± SD serum concentrations of IL-2 (**a**), IL-6 (**b**), IL-10 (**c**), TNF-α (**d**), and IFN-γ (**e**) from all evaluable patients during the first treatment week of each of the first three cycles. Corresponding patient numbers are shown in Additional file 5

Serum samples from all 36 treated patients were analyzed for the release of IL-2, IL-4, IL-6, IL-10, TNF-α, and IFN-γ during the first week of treatment cycles 1–3. Before blinatumomab infusion, all measured cytokines were below the LOD of 20 pg/mL. Within the first hours after infusion start in cycle 1, levels of IL-2, IL-6, IL-10, TNF-α, and IFN-γ, but not IL-4, increased rapidly, reaching peak mean concentrations after 2 h (TNF-α), 6 h (IL-2), or 24 h (IL-6, IL-10, IFN-γ) before returning to baseline levels at the end of the 1st week (Fig. 5a‒e). Cytokines with the highest serum levels measured during cycle 1 were IL-6, IL-10, and IFN-γ. IL-10 was the only cytokine that was markedly elevated in treatment cycles 2 and 3.

### Mitigation of cytokine release using blinatumomab step-dosing

**Fig. 6:**
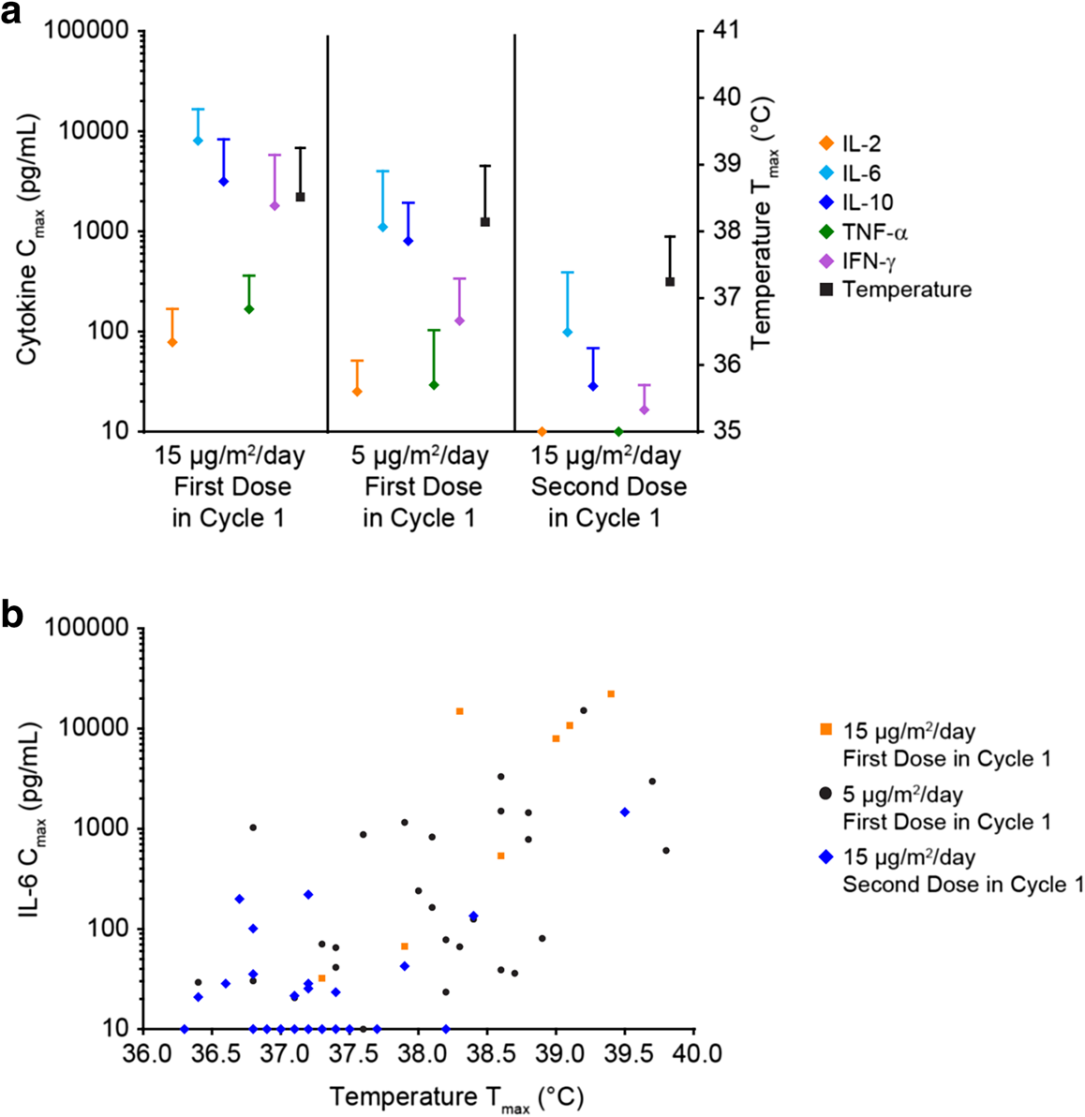
Mitigation of cytokine release by blinatumomab step-dosing. **a** Mean peak serum concentrations (C_max_) ± SD of cytokines IL-2, IL-6, IL-10, TNF-α, IFN-γ and mean peak body temperature (T_max_) ± SD in patients who received blinatumomab doses of either 5 or 15 µg/m^2^/day as first dose during treatment week 1, or 15 µg/m^2^/day as second dose in week 2. **b** C_max_ of IL-6 and T_max_ in patients receiving different blinatumomab doses during week 1 or 2. *Orange* 15 µg/m^2^/day flat dose; *black* 5 µg/m^2^/day first dose in cycle 1; *blue* 15 µg/m^2^/day second dose in cycle 1. Corresponding patient numbers are shown in Additional file 6

Mean peak cytokine concentrations were also studied in correlation with the starting dose in cycle 1 (5 or 15 µg/m^2^/day as first dose; 15 µg/m^2^/day as second dose). Cytokine levels (IL-2, IL-6, IL-10, TNF-α, and IFN-γ) were higher in patients who received a starting dose of 15 µg/m^2^/day than those who received 5 µg/m^2^/day (Fig. 6a). However, patients receiving 5 µg/m^2^/day as the first blinatumomab dose in cycle 1 followed by 15 µg/m^2^/day as second dose 1 week later showed a lower cytokine secretion at both dose levels, compared with patients who did not receive step-dosing. At the dose step to 15 µg/m^2^/day, cytokine levels were even lower than at 5 µg/m^2^/day (Fig. 6a). Analysis of peak body temperatures showed higher mean temperatures for those patients who started treatment at a higher blinatumomab dose compared with those who received step-dosing (Fig. 6a). Higher peak body temperatures was associated with higher IL-6 peak concentrations, especially in patients who received blinatumomab flat dosing of 15 µg/m^2^/day (Fig. 6b).

### Blinatumomab-induced cytokine and granzyme B release

**Fig. 7:**
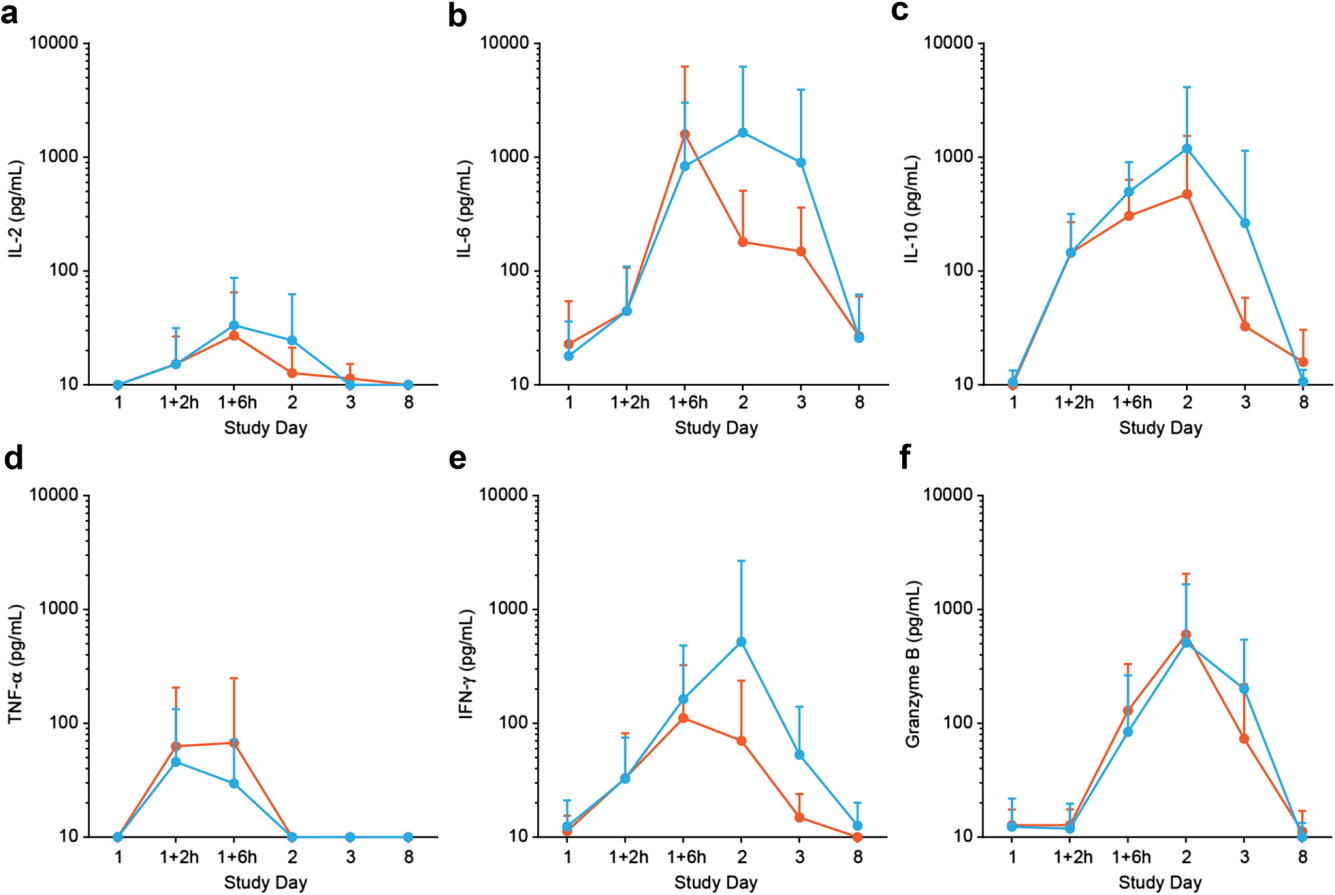
Cytokine and granzyme B release during the first treatment week in responding and nonresponding patients. Patients were stratified into responders (*blue*) and nonresponders (*red*) based on CR/CRh response within the first two treatment cycles. Mean ± SD serum concentrations of IL-2 (**a**), IL-6 (**b**), IL-10 (**c**), TNF-α (**d**), IFN-γ (**e**), and granzyme B (**f**) from all evaluable patients during the first treatment week are depicted on a logarithmic scale. Corresponding patient numbers are shown in Additional file 7

We compared mean serum concentrations of cytokines and the release of the T-cell effector molecule granzyme B in responders and nonresponders during the first treatment week of cycle 1. Patients who achieved CR/CRh appeared to have had higher mean serum concentrations of IL-6, IL-10, and IFN-γ, with peak maxima reached within the first 24 h after blinatumomab infusion start (Fig. 7b, c, e). In contrast, mean serum concentrations of IL-2 and TNF-α were not different between both patient groups (Fig. 7a, d). Similarly, analysis of mean granzyme B levels in responders and nonresponders during the first treatment week showed a comparable granzyme B kinetic for both groups, with transient elevation of granzyme B levels within the first 24 h after blinatumomab infusion start (Fig. 7f).

## Discussion

This is the first study reporting in detail on changes in laboratory and pharmacodynamic parameters in response to blinatumomab treatment in the setting of relapsed/refractory ALL using data from an exploratory dose-finding phase II study. Changes in most of the evaluated liver and first-dose parameters were characterized by rapid elevations immediately after infusion start and return to baseline at the end of the cycle. Specifically, ALT and AST were moderately elevated after infusion start (<5 times the normal range) but decreased to baseline within the first cycle. Mild to moderately elevated ALT and AST are the most frequently observed clinical transaminase alterations [19, 21] and are not uncommon in response to medications as described for various other drugs, such as statins and methotrexate [22]. The observed transiently elevated levels of LDH, which may indicate tissue damage, and D-dimer, a coagulation marker [23], might be associated with blinatumomab-induced B-cell lysis and adhesion of lymphocytes and platelets to blood vessel endothelium. Similar changes in laboratory parameters, including elevated GGT, have been described for patients with B-cell chronic lymphocytic leukemia receiving the anti-CD20 monoclonal antibody rituximab [19, 24, 25]. Importantly, the increases in liver and first-dose parameters, including CRP, a marker of infection and inflammatory processes [26], in response to blinatumomab were transient and reversible, and did not result in treatment interruptions or discontinuations. Furthermore, pronounced elevations in those parameters appear to occur early in the course of treatment when most patients with relapsed/refractory ALL receive blinatumomab in the hospital (week 1 of cycle 1), which would allow for careful monitoring and management, especially with respect to treatment interruptions.

Blinatumomab activates T cells only in the presence of CD19^+^ target cells. Incubation of isolated T cells in the absence of target cells even at saturating blinatumomab concentrations has not been shown to cause expression of activation markers CD69, CD25, or release of cytokines [27–29]. Therefore, blinatumomab-induced PD effects in patients with ALL or NHL are most likely caused by activation of T cells after binding of accessible CD19^+^ target cells and formation of a cytolytic synapse. Several PD effects have been reported in studies with blinatumomab in patients with MRD-positive ALL [6, 10] or relapsed/refractory NHL [20]. In both settings, B-cell depletion, T-cell redistribution, and cytokine elevation were most pronounced during the first days after the start of blinatumomab infusion. The qualitative pattern of PD effects (such as transient T-cell redistribution) that occur shortly after infusion start in each treatment cycle, or after each dose step, was comparable across those studies. In patients with relapsed/refractory ALL, peripheral B cells rapidly declined within the first two treatment days, independent of clinical response to blinatumomab. This pattern of B-cell depletion is similar to that observed in patients with MRD-positive ALL or relapsed/refractory NHL, providing an early marker of blinatumomab activity [10, 20]. In patients with relapsed/refractory ALL there was an association between T-cell expansion and clinical response to blinatumomab during cycle 1, and T-cell expansion has been associated with long-term survival in this setting [9]. T-cell counts above baseline may be caused by a delayed return of T cells from target tissue back into peripheral blood, by T-cell proliferation, or both. Increased CD3^+^ T-cell counts of responding patients coincided with expanding CD4^+^ and CD8^+^ T cells and memory T cells, especially during treatment cycle 1. Similarly, T-cell expansion has also been described for MRD-positive ALL and relapsed/refractory NHL. In the MRD-positive ALL study, T-cell expansion above baseline was limited to the first treatment cycle and both CD4^+^ and CD8^+^ T cells contributed to T-cell expansion, but neither was associated with response [10, 20]. The present analysis suggests an association between T-cell expansion and clinical response to blinatumomab in relapsed/refractory ALL; however, the results are based on small numbers of patients. Appropriately designed larger studies are required to validate T-cell expansion as a biomarker for clinical response to blinatumomab treatment in this setting.

The redistribution kinetics of peripheral T cells in patients with relapsed/refractory ALL were also comparable to previously published data. As described for MRD-positive ALL and relapsed/refractory NHL [3, 10, 20], peripheral T cells rapidly disappeared from circulation within the first day of infusion before recovering to baseline after approximately 1 week. This T-cell disappearance is most likely a consequence of increased T-cell adhesion to blood vessel endothelium as evident from the affinity shift in the activated cell adhesion molecule LFA-1 on T cells. Simultaneously, the early activation marker CD69 was upregulated on both CD4^+^ and CD8^+^ T cells, suggesting that T cells were activated upon encounter with peripheral B cells. Involvement of both CD4^+^ and CD8^+^ T cells in redistribution and activation has been observed in all clinical studies with blinatumomab conducted to date [4, 10, 20].

The effector molecule granzyme B is stored in secretory vesicles of cytotoxic T cells and is released upon formation of a blinatumomab-mediated cytolytic synapse between a T and B cell [30]. Our data show that in patients with relapsed/refractory ALL, peak levels of granzyme B during the 1st week of blinatumomab infusion were not associated with a clinical response, but its rapid appearance in serum provides additional evidence of the biologic activity of blinatumomab.

This is the first study reporting on the distribution kinetics of neutrophils and thrombocytes in patients with relapsed/refractory ALL in response to blinatumomab. Both responders and nonresponders had low platelet counts before infusion start. Thrombocytopenia is frequently associated with polychemotherapy, a common treatment for relapsed/refractory ALL, and bone marrow infiltration by blast cells, thus reflecting the disease state. After start of blinatumomab treatment, thrombocyte counts decreased further during the first treatment days before recovering and even achieving normal levels in the responder group at the end of cycle 1. The initial platelet redistribution coincided with the described T-cell redistribution and might be explained by an increased adhesiveness of activated blood vessel endothelium, leading to platelet adhesion and disappearance from the circulation. Surprisingly, neutrophils showed the opposite behavior, with mean cell counts initially increasing after infusion start. It can be speculated that this neutrophil spike, which coincides with T-cell redistribution, is caused by other lymphocytes and platelets displacing neutrophils from the blood vessel endothelium, thus releasing them into the circulation.

Blinatumomab treatment frequently causes fever and can cause rare events of CRS or cytokine storm, a consequence of extensive cytokine release following blinatumomab-induced T-cell activation [6, 7]. Transient elevations of cytokines during the first treatment week have been described for patients with MRD-positive ALL [10]. Those elevations were limited to cycle 1 and were not associated with clinical response. The overall pattern of released cytokines in the present study was similar; however, the anti-inflammatory cytokine IL-10 was also detectable in cycles 2 and 3, possibly because of the larger tumor load in patients with relapsed/refractory ALL, compared with MRD-positive ALL. The magnitude of IL-6, IL-10, and IFN-γ release was slightly higher in patients who achieved CR/CRh than in nonresponders. It may be speculated that increased IL-10 levels, or even IL-6 or IFN-γ levels, in the responder group were an early sign of clinical response. However, data from a larger number of evaluable patients are required to establish an association between cytokine release and the antileukemic activity of blinatumomab. A possible association may be supported though by the observation that IL-10 does not only possess anti-inflammatory but also immune-stimulatory properties. This has recently been described for pegylated recombinant IL-10, which stimulated the activation, expansion, and cytotoxicity of tumor-infiltrating CD8^+^ T cells, while increased levels of immune stimulatory cytokines and antitumor activity were observed in patients with solid tumors who received pegylated recombinant IL-10 [31].

Blinatumomab first-dose effects, including CRS, in patients with relapsed/refractory ALL have been successfully mitigated using stepwise dosing [6]. We have shown for the first time that stepwise dosing resulted in a reduction in cytokine release and in lower body temperature, which explains the better tolerability of this dosing regimen compared with flat dosing. Patients with relapsed/refractory ALL who receive stepwise dosing, especially at the beginning of therapy, experienced few cytokine-associated clinical symptoms, such as CRS or cytokine storm [6, 7]. Our data also support the administration of stepwise dosing when restarting blinatumomab treatment after CRS-associated infusion interruption once the CRS event has resolved.

## Conclusions

This is the first detailed PD analysis of patients with relapsed/refractory ALL who received continuous IV infusion of the CD19-targeting BiTE^®^ antibody construct blinatumomab. The findings identify for future evaluation possible biomarkers for clinical response in a disease setting characterized by high tumor load and poor prognosis, and provide a clear rationale for blinatumomab step-dosing.

## Additional files

**Additional file 1.** Available patient numbers (N) for analysis of liver and first-dose parameters in Fig. 1.

**Additional file 2.** Available patient numbers (N) for analysis of distribution profiles of neutrophils, thrombocytes, T cells and B cells in Fig. 2.

**Additional file 3.** Available patient numbers (N) for analysis of T-cell redistribution, LFA activation, and CD69 upregulation in Fig. 3.

**Additional file 4.** Available patient numbers (N) for analysis of distribution profiles of CD8^+^ and CD4^+^ T cells and subsets in Fig. 4.

**Additional file 5.** Available patient numbers (N) for analysis of cytokine profiles in Fig. 5.

**Additional file 6.** Available patient numbers (N) for analysis of cytokine and body temperature peak maxima in Fig. 6.

**Additional file 7.** Available patient numbers (N) for analysis of cytokine and granzyme B profiles in Fig. 7.

## Abbreviations

ALL: acute lymphoblastic leukemia
ALT: alanine aminotransferase
ANC: absolute neutrophil count
AP: alkaline phosphatase
AST: aspartate aminotransferase
BiTE^®^: bispecific T-cell engager
C_max_: maximum or peak concentration
CR: complete remission
CRh: complete remission with partial hematologic recovery
CRP: C-reactive protein
CRS: cytokine release syndrome
C_ss_: steady state concentration
FACS: fluorescence-activated cell sorter
GGT: gamma–glutamyl transferase
Hb: hemoglobin
IFN: interferon
IL: interleukin
LDH: lactate dehydrogenase
LLOQ: lower limit of quantification
LOD: limit of detection
MRD: minimal residual disease
NHL: non-Hodgkin’s lymphoma
PD: pharmacodynamics
PK: pharmacokinetics
SD: standard deviation
T_CM_: central memory T cells
T_EM_: effector memory T cells
T_EMRA_: CD45RA^+^ effector memory T cells
TNF: tumor necrosis factor
ULN: upper limit of normal
