# Supplementary material for: Changes in clinical laboratory parameters and pharmacodynamic markers in response to blinatumomab treatment of patients with relapsed/refractory ALL
Source: Exp Hematol Oncol. 2017 May 18;6:14. doi: 10.1186/s40164-017-0074-5 (PMC5437652; doi:10.1186/s40164-017-0074-5)
Supplement: Supplementary file 2 — Additional file 2. Available patient numbers (N) for analysis of distribution profiles of neutrophils, thrombocytes, T cells and B cells in Fig. 2. [file 40164_2017_74_MOESM2_ESM.docx]

**ADDITIONAL FILE 2**

**Available patient numbers (N) for analysis of distribution profiles of neutrophils, thrombocytes, T cells and B cells in Figure 2.**


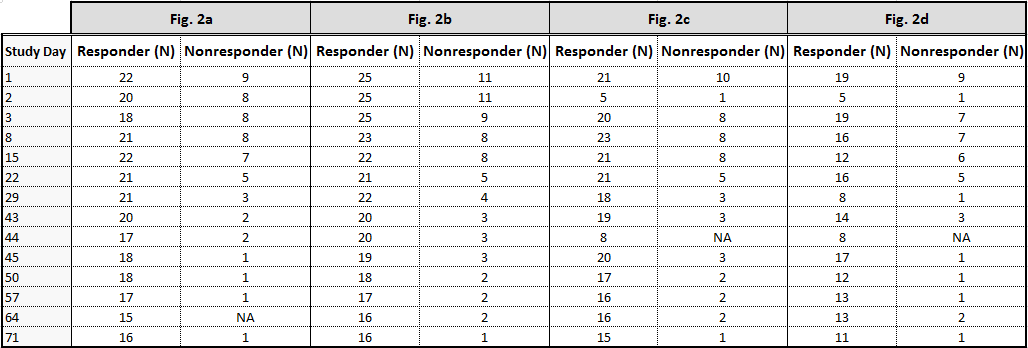


NA, no data available
