# Supplementary material for: Changes in clinical laboratory parameters and pharmacodynamic markers in response to blinatumomab treatment of patients with relapsed/refractory ALL
Source: Exp Hematol Oncol. 2017 May 18;6:14. doi: 10.1186/s40164-017-0074-5 (PMC5437652; doi:10.1186/s40164-017-0074-5)

**ADDITIONAL FILE 4**

**Available patient numbers (N) for analysis of distribution profiles of CD8^+^ and CD4^+^ T cells and subsets in Figure 4.**


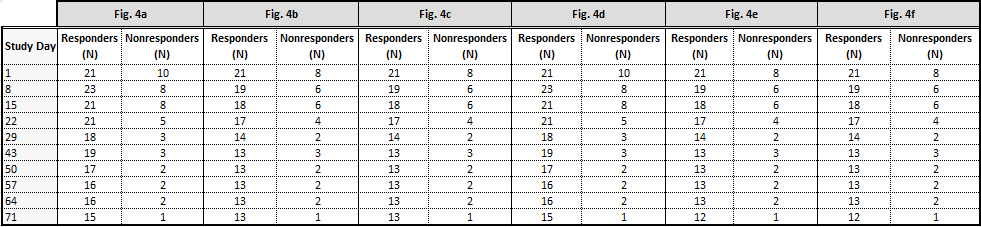

Supplement: Supplementary file 4 — Additional file 4. Available patient numbers (N) for analysis of distribution profiles of CD8+ and CD4+ T cells and subsets in Fig. 4. [file 40164_2017_74_MOESM4_ESM.docx]
