# Supplementary figures and images for: Changes in clinical laboratory parameters and pharmacodynamic markers in response to blinatumomab treatment of patients with relapsed/refractory ALL
Source: Exp Hematol Oncol. 2017 May 18;6:14. doi: 10.1186/s40164-017-0074-5 (PMC5437652; doi:10.1186/s40164-017-0074-5)

**ADDITIONAL FILE 5**

**Available patient numbers (N) for analysis of cytokine profiles in Figure 5.**


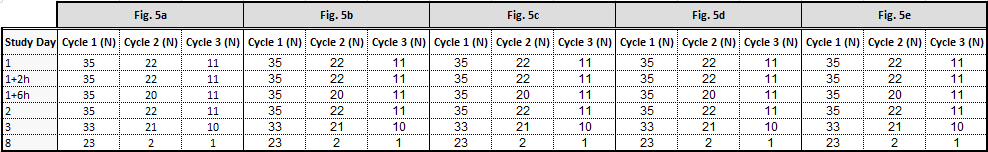

Supplement: Supplementary file 5 — Additional file 5. Available patient numbers (N) for analysis of cytokine profiles in Fig. 5. [file 40164_2017_74_MOESM5_ESM.docx]
