# Supplementary material for: Changes in clinical laboratory parameters and pharmacodynamic markers in response to blinatumomab treatment of patients with relapsed/refractory ALL
Source: Exp Hematol Oncol. 2017 May 18;6:14. doi: 10.1186/s40164-017-0074-5 (PMC5437652; doi:10.1186/s40164-017-0074-5)

**ADDITIONAL FILE 7**

**Available patient numbers (N) for analysis of cytokine and granzyme B profiles in Figure 7.**


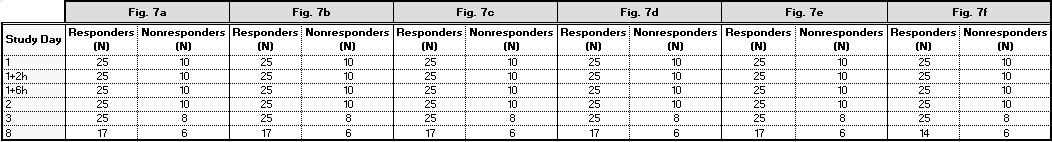

Supplement: Supplementary file 7 — Additional file 7. Available patient numbers (N) for analysis of cytokine and granzyme B profiles in Fig. 7. [file 40164_2017_74_MOESM7_ESM.docx]
